# Dual-flow network with attention for autonomous driving

**DOI:** 10.3389/fnbot.2022.978225

**Published:** 2023-01-09

**Authors:** Lei Yang, Weimin Lei, Wei Zhang, Tianbing Ye

**Affiliations:** ^1^School of Computer Science and Engineering, Northeastern University, Shenyang, China; ^2^DAMO Academy, Alibaba Group, Hangzhou, China; ^3^Engineering Research Center of Security Technology of Complex Network System Ministry of Education, Shenyang, China

**Keywords:** autonomous driving, network architecture, artificial intelligence, CARLA simulator, attention, visual navigation, deep neural network

## Abstract

We present a dual-flow network for autonomous driving using an attention mechanism. The model works as follows: (i) The perception network extracts red, blue, and green (RGB) images from the video at low speed as input and performs feature extraction of the images; (ii) The motion network obtains grayscale images from the video at high speed as the input and completes the extraction of object motion features; (iii) The perception and motion networks are fused using an attention mechanism at each feature layer to perform the waypoint prediction. The model was trained and tested using the CARLA simulator and enabled autonomous driving in complex urban environments, achieving a success rate of 74%, especially in the case of multiple dynamic objects.

## 1. Introduction

Autonomous driving, especially in a complex environment, remains a challenging subject. However, with the evolution of deep networks, breakthroughs have been made in autonomous driving techniques using images as input (Chen et al., 2015; Dosovitskiy et al., 2017; Liang et al., 2018; Li et al., 2018; Sauer et al., 2018). These studies have been broadly based on using images as input, extracting features through deep neural networks, and then converting feature mapping to the vehicle's low-level commands. However, the image is only a spatial representation of the environment *I*(*x, y*), so we lose the temporal dimension of the video, which is also crucial for the understanding of motion. Therefore, we attempt to add the consideration of temporal dimension and introduce the method in the domain of video recognition to autonomous driving for completing the extraction of space–time features of the surrounding environment. This raises the first question: *Are the spatial and temporal characteristics of the video equally important?* Weiss et al. (2002) showed that the human retina had a different sensitivity to understanding scene and moving objects, where scene understanding needs to be precise but changes slowly, while the perception of motion of corresponding objects is the opposite. Motivated by Weiss et al. (2002) and Feichtenhofer et al. (2019), we ensure that the perception network maintaining the high-resolution spatial features of the image, while the motion network focuses on real time using a more lightweight network. The ablation experiments (shown in **Table 4**) demonstrated that a ratio of 8:1 between the number of feature channels of the perception network and the motion network worked best.

The perceptual and motion features of the video are a whole in themselves, which leads to the second question to be solved: *How can we integrate the perceptual features with the motion features?* The attention mechanism has received more focus in recent years owing to its success in natural language processing (NLP), and there have been increased developments in the image domain as well (Dosovitskiy et al., 2020; Liu et al., 2021). Inspired by these studies, we ensure that the dual-flow network performs attention learning in each feature layer (except the first layer) and that the high-resolution perception network aligns motion features to more accurately perceive the motion of objects in a complex environment, such as pedestrian intrusion.

**Figure 1:**
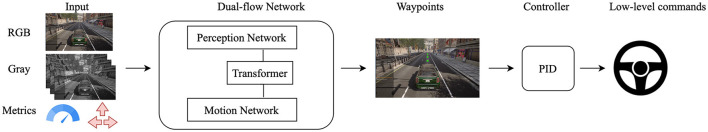
Overview of the structure, including the video from the camera, vehicle speed, and high-level navigation commands as input, using a dual-flow network to extract features and output the prediction of waypoints, which are converted to the vehicle's low-level commands through the proportional-integral-derivative (PID) controller.

The overall model structure is shown in Figure 1. The vehicle's front camera is used as input, and the features are extracted through a dual-flow network to perform the waypoint prediction, which is finally mapped to the vehicle's low-level commands (steering, throttle, and brake) by proportional–integral–derivative (PID) controllers. As far as we know, the attention-based dual-flow network is being used for the first time in autonomous driving. Our main contributions are as follows: (1) Proposing the dual-flow network to extract space–time features from video; (2) Using an attention mechanism to fuse space–time features in the dual-flow network; and (3) Conducting experiments to demonstrate the network's effectiveness. Using the CARLA (Dosovitskiy et al., 2017) simulator, we can adapt the model to the complex urban environment for autonomous driving, with a success rate of 74%.

## 2. Related work

### 2.1. Autonomous driving

Prior approaches for autonomous driving with computer vision are classified into three groups: modular pipelines (MP), imitation learning (IL) (Hussein et al., 2017), and direct perception (DP) (Gibson, 2014).

Modular pipelines are the relatively more traditional and longer studied approach. The task is generally divided into three sub-modules: a perception module, a planning module, and a control module. Among them, the perception module is the core of the whole system and is commonly used for the identification of lanes, fences, dynamic objects, and other hazards, providing inputs for subsequent modules. Geiger et al. (2013) investigated the various sub-tasks. Felzenszwalb et al. (2010) and Lenz et al. (2011) detected vehicles and lanes, respectively. Lin et al. (2017) attempted to segment the image. The outputs of the perception modules of these methods cannot be used directly for autonomous driving tasks and need to be subsequently mapped. As MP relies on many intermediate feature representations, multiple intermediate feature accuracies limit the accuracy of the entire system, which is more challenging in complex environments.

Imitation learning is a bionic approach also called behavioral cloning, which maps the raw sensor inputs directly to the vehicle's low-level control commands, and is an end-to-end learning approach. Our model also belongs to this category. First, Pomerleau (1988) and Muller et al. (2005) used the camera as the main input and used a neural network to predict the related actions. Codevilla et al. (2018) used high-level navigation commands to eliminate ambiguity at intersections for autonomous driving, opening up a new stage of navigation. Li et al. (2018) enabled better generalization of the model by adding branches that assist in predicting image depth and image segmentation. Codevilla et al. (2019) added speed prediction while exploring some limitations of autonomous driving. Chen et al. (2020) proposed a dual-network structure of “teacher” and “student” using the knowledge distillation method and optimized the “student” network through the “teacher” network, achieving good results. A multimodal fusion study by Xiao et al. (2020) was attempted and demonstrated that early feature fusion helped in feature learning. Research related to our work (Prakash et al., 2021) incorporated both image and light-detection-and-ranging (LiDAR) modalities with an attention mechanism, while our model focuses on the mining of video space–time features. Both the modality and the network architecture are different.

Direct perception is an intermediate form of MP and IL, which decompose the autonomous driving task into several key metrics, enhancing interpretation without relying on an intermediate representation of the environment. Chen et al. (2015) attempted to map the camera input to 13 metrics related to the final decision. Sauer et al. (2018) mapped images directly to six sub-tasks emergency stop, red light, speed marker, vehicle distance, steering angle, and lane departure, and those metrics were converted to throttle and brake commands by rules. The DP approach requires a manual setting of prediction sub-tasks, which is difficult to accomplish in complex environments.

### 2.2. Space–time features

In the image domain, convolutional neural networks (CNN) have dominated, including AlextNet (Krizhevsky et al., 2012), VGG (Simonyan and Zisserman, 2014b), ResNet (Szegedy et al., 2017), and ConvNeXt (Liu et al., 2022), and have now become the base networks for extracting image features.

Recently, there have also been some major developments in the video domain. The network of two-dimensional architectures includes the following: DeepVideo (Karpathy et al., 2014), TwoStreamNet (Simonyan and Zisserman, 2014a) and TSN (Wang et al., 2016), and a series of studies. The network of three-dimensional architectures includes the following: C3D (Tran et al., 2015), I3D (Carreira and Zisserman, 2017), and a series of studies. Transformer network architecture contains TimesTransformer (Bertasius et al., 2021) and ViT (Girdhar et al., 2019).

In the temporal dimension of video, the optical flow defines the movement of objects in an image, specifically the amount of movement of pixels representing the same object in one frame of a video image to the next frame. The most classical traditional optical flow algorithm is that proposed by Lucas and Kanade (1981), which has been widely used given its luminance invariance assumption and neighborhood optical flow similarity assumption, and has been integrated into the OpenCV library. Considering the computational inefficiency of traditional optical flow algorithms, in recent years, people have tried to estimate the optical flow using deep neural networks (DNNs), represented by algorithms such as FlowNet2 (Ilg et al., 2017). In the field of autonomous driving, optical flow also has a wide range of applications. Gern et al. (2002) used optical flow to do lane recognition under adverse weather conditions. In Lieb et al. (2005), the optical flow was used within a road-following algorithm that allows for identifying the road. In Okafuji et al. (2015), steering learning was performed in in-car driving. Camus (1995) performed obstacle avoidance using optical flow. In Capito et al. (2020), the visual potential field was calculated using optical flow vectors for obstacle avoidance. Optical flow distillation paradigms have also recently emerged for application in the field of autonomous driving (Rashed et al., 2019). However, instead of using optical flow as input, our motion network tries to have the network automatically learn motion-related features and then fuse them with the individual feature layers of the perceptual network features. The network features we learn are more related to autopilot-related motion features and are not different from the optical flow feature as a whole (e.g., large changes in building illumination are not relevant for autopilot features).

Our dual-flow network is motivated by a two-stream net and slow-fast net, but has a different input and network architecture from either of them. Our inputs contain RGB images and continuous video frames (grayscale maps), while the feature network uses a modified ResNet network to maintain high resolution and guarantee feature extraction of environmental details and motion.

### 2.3. Attention mechanisms

An attention (Vaswani et al., 2017) mechanism can learn the attention of features dynamically based on different predicted targets. The earlier applications of attention mechanisms were mainly in the fields of NLP, but recently they have evolved significantly in both image and video domains, from the early squeezeNet (Iandola et al., 2016) and non-local (Wang et al., 2018) to transformer. An attention mechanism is distinctly different in the three domains of NLP, computer vision, and image recognition. The first two are closely related to the temporal dimension, while the input of the third one is a static picture without a clear sequence. Our model uses the attention mechanism in the field of computer vision to accomplish the fusion of dual-flow network features.

## 3. Method

**Figure 2:**
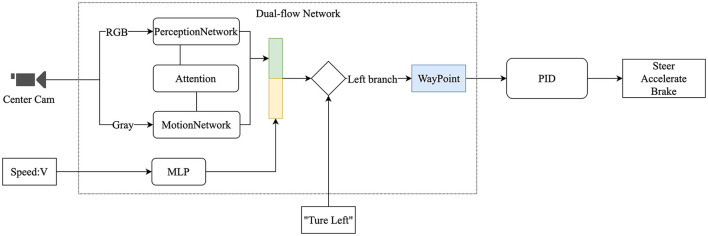
Flow chart of the overall system. The process has three steps: (1) Using the vehicle's front camera and speed as inputs, the low-sampling RGB image is used as input to the perception network, and the high-sampling grayscale images are used as input to the motion network; (2) The dual-flow network overlapping the high-level navigation commands performs the prediction of waypoints; and (3) The final output of the vehicle's low-level control commands is produced through the PID controllers.

The overall system is an end-to-end learning framework, as shown in Figure 2. The RGB images sampled at low sample rates are used as input to the perception network, and the grayscale images at high sample rates are used as input to the motion network. The dual-flow network extracts features and fuses them through an attention mechanism to output features, concatenates the speed features, and then completes the selection of the corresponding branches according to the high-level navigation commands to output the waypoints. Finally, the waypoints are mapped to the low-level vehicle commands by the PID controllers. The details are described in later sections.

### 3.1. Problem setting

Autonomous driving can be seen as a bionic learning or behavior cloning process. The process is seen as a supervised learning problem, where the behavior of an expert is imitated through a deep network. First, the data of the expert-driven vehicle are collected in the environment to form the training data set $D={\left\{\left(O_{i},W_{i}\right)\right\}}_{i=1}^{N}$, where *O*_*i*_ denotes the ith observation of the vehicle to the environment, and *W*_*i*_ denotes the ith ground-truth waypoint. The goal is to fit a function *F* with θ parameters through a neural network such that the output of F is as similar as possible to that of *W*. The DNN represents the mapping function *F*_θ_. The learning procedure aims to continuously optimize θ so that the loss value is minimized.


(1) $$\begin{array}{l} {Minimize}_{\theta }\underset{i}{\sum }L\left(F\left(O_{i};\theta \right),W_{i}\right) \end{array}$$

where *W*_*i*_ is the ith ground-truth waypoint, *F* is the waypoint prediction network with the θ parameter, and *O*_*i*_ denotes the ith observation of the vehicle to the environment. *L* is the loss function for the prediction of waypoints and the ground-truth waypoints.

Each episode has an end position, marked as *D*_*g*_, while *D*_*g*_ determines the high-level navigation commands at the intersection *D*_*g*_ = {*C*_*i*_, …, *C*_*m*_} where *C*_*i*_ denotes the ith high-level navigation command in one episode at the intersection, which eliminates the ambiguity of the intersection and has been demonstrated in Codevilla et al. (2018) and subsequent studies to be important. Thus, we also use *C*_*i*_ as a part of the training sample. The final input is represented as *O*_*i*_ = {(*I, C, V*)}_*i*_, where *I* denotes the image input, *C* ∈ {*Left, Right, Straight*} denotes the high-level navigation commands, and *V* denotes the vehicle speed. The final optimization function is derived from Formula 1.


(2) $$\begin{array}{l} \theta^{*}={argmin}_{\theta }\underset{i}{\sum }L\left(F\left({\left(I,C,V\right)}_{i};\theta \right),W_{i}\right) \end{array}$$

where *F* is the waypoint prediction network with θ parameter, and *I, C, V* are the inputs. *I* represents the image from the camera, *C* represents the high-level navigation commands, and *V* is the vehicle speed. *W*_*i*_ is the ith ground-truth waypoint. θ^*^ represents the final neural network parameters to be learned.

The network outputs the predicted waypoints which are then translated by the PID controller into the low-level control commands for steering, throttling, and braking, expressed as follows:


(3) $$\begin{array}{l} \begin{array}{c} U_{i}=PID\left(F\left(O_{i};\theta^{*}\right)\right) \end{array} \end{array}$$

where *F* presents the network with θ parameters and predicts the waypoints using *O* as inputs. *O*_*i*_ is the ith observation of the environment, specifically containing the camera, vehicle speed, and high-level navigation commands. *PID* is the PID controller function, which does the mapping of waypoints to low-level commands. Our model uses two PID controllers: lateral PID and longitudinal PID. *U*_*i*_ is the ith specific control command to manipulate the vehicle, *U*_*i*_ ∈ {*Steer, Throttle, Brake*}.

### 3.2. Perception and plan module

**Figure 3:**
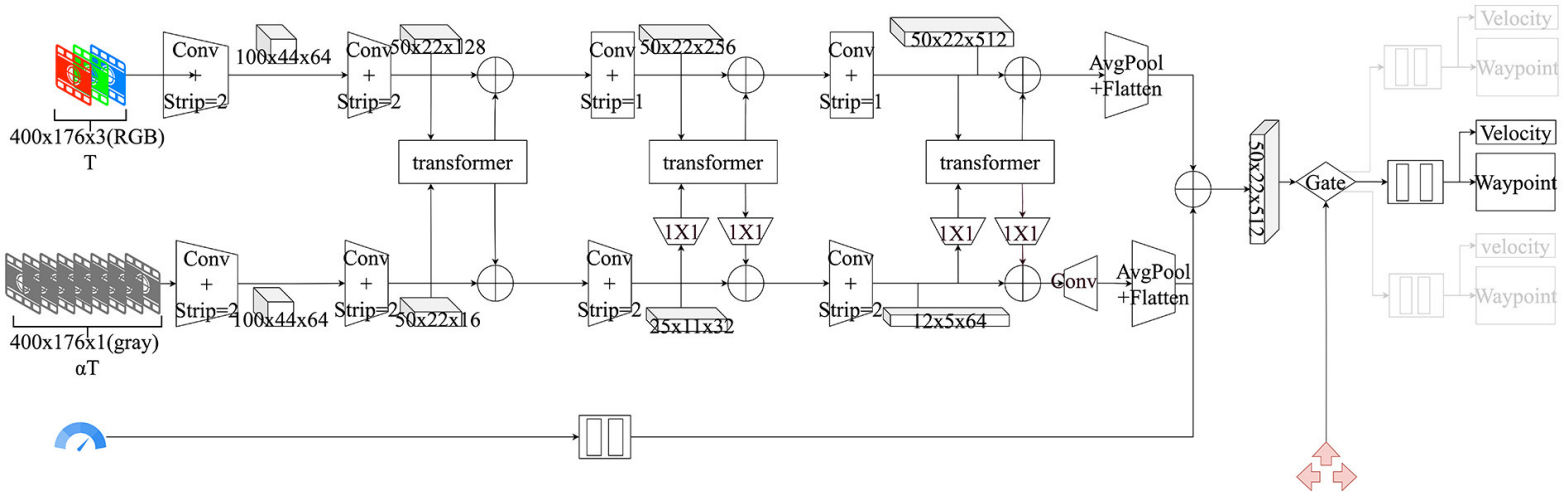
Architecture of dual-flow network. The first row is the perception network using an RGB image as input, the second row is the motion network using a sequence gray image as input, and the third row presents the input of the velocity. *T* denotes the sampling rate of images per second, and α denotes the ratio of the image sampling rate of the perception network and motion network.

The perception and plan module used by the model specifically refer to the dual-flow network, as shown in Figure 3. *T* denotes the sampling rate of images per second, and α represents the ratio of the image sampling rate of the perception network and motion network. The dual-flow network is the core and innovative point that does the mapping from the original image and dashboard metrics to the waypoints. The design of the dual-flow network is inspired by the study of retinal neural cells (Weiss et al., 2002). Specifically, both the perception network and motion network use the video captured by the camera as input; however, the sampling rate is different. Finally, the features are fused using the attention mechanism.

#### 3.2.1. Input and output

The input is divided into images, speed, and high-level navigation commands. The dual-flow network is fed with the video captured by the vehicle's front camera, but at different sampling rates. The perception network takes as the input an RGB image with the *T* sampling rate, labeled as *I*_*rgb*_: = *T*, and the motion network takes as the input a gray image with the α*T* sampling rate, denoted as *I*_*gray*_. The ratio of input is represented as


(4) $$\begin{array}{l} I_{rgb}=\alpha I_{gray} \end{array}$$

where α represents the ratio of the image sampling rate of the perception network and motion network; in our model, *T* = 3, α = 8, that is, there are three images per second for the perception network input, corresponding to 8 × 3 images for the motion network. The speed input is based on the vehicle dashboard speed and is normalized by $V=\frac{V_{current}}{V_{max}}$, where *V*_*current*_ represents the current speed of the vehicle, and *V*_*max*_ is the maximum speed limited by the model. When the value of *V* = 0, it means stationary, and when the value is 1, it means the maximum speed. The high-level navigation command is denoted by the symbol *C*_*i*_ ∈ {*Left, Right, Straight*}.

The output represents the predicted waypoints. These waypoints are used as inputs to the PID controller and are converted to the vehicle's low-level control commands.

#### 3.2.2. Network architecture

The dual-flow network is not limited to a specific backbone network architecture to be used. Considering the ease of deployment and the broadness, we adopt the RestNet-34 backbone network and make important improvements to it, as shown in the following Table 1.

**Table 1:** Details of the structure of the dual-flow network framework.

**Stage**	**Perception net**	**Motion net**	**Output size *H*×*W*×*C***
Input	-	-	*PNet*:400 × 176 × 3, *MNet*:400 × 176 × 8
Conv1	7 × 7, 64, *s* = 2	7 × 7, 64, *s* = 2	*PNet*:200 × 88 × 64, *MNet*:200 × 88 × 64
Pool1	3 × 3, *maxpool, s* = 2	3 × 3, *maxpool, s* = 2	*PNet*:100 × 44 × 64, *MNet*:100 × 44 × 64
Stage2	$\left[\begin{array}{c} 3\times 3,64\\ 3\times 3,64 \end{array}\right]\times 3$	$\left[\begin{array}{c} 3\times 3,8\\ 3\times 3,8 \end{array}\right]\times 3$	*PNet*:100 × 44 × 64, *MNet*:100 × 44 × 8
Stage3	$\left[\begin{array}{c} 3\times 3,128\\ 3\times 3,128 \end{array}\right]\times 4,s=1$	$\left[\begin{array}{c} 3\times 3,16\\ 3\times 3,16 \end{array}\right]\times 4$	*PNet*:50 × 22 × 128, *MNet*:50 × 22 × 16
Stage4	$\left[\begin{array}{c} 3\times 3,256\\ 3\times 3,256 \end{array}\right]\times 6,s=1$	$\left[\begin{array}{c} 3\times 3,32\\ 3\times 3,32 \end{array}\right]\times 6$	*PNet*:50 × 22 × 256, *MNet*:25 × 11 × 32
Stage5	$\left[\begin{array}{c} 3\times 3,512\\ 3\times 3,512 \end{array}\right]\times 3,s=1$	$\left[\begin{array}{c} 3\times 3,64\\ 3\times 3,64 \end{array}\right]\times 3$	*PNet*:50 × 22 × 512, *MNet*:12 × 5 × 64
Deconv	-	3 × 3, *s* = 4, *p* = 1, *outpadding* = 5, *c* = 512	*PNet*:50 × 22 × 512, *MNet*:50 × 22 × 512
Avg + sum	1 × 1	1 × 1	1 × 1 × 512

The bold digit indicates network improvement points, where red and green values correspond to the number of channel and the stripe size, respectively. The perception network maintains the resolution from stage 3. The motion network reduces residual channels to one-eighth of their original size.

The perception network's strip from stage 3 in the residual module is set to 1, so the resolution is not reduced, and the final output is a *P*_*o*_ = (50 × 22 × 512) feature vector. Intuitively, high-resolution features help recognize image details.

The motion network considers the motion of the object as a whole and does not require pixel-level differentiation. Compared with the perception network, the motion network has a number of channels that is reduced by a factor of 8, and the resolution is reduced by a factor of 32, resulting in a final output *M*_*o*_ = (12 × 5 × 32). The motion network helps to provide sensitivity to motion and improves the detection speed, in agreement with Weiss et al. (2002)'s conclusion.

Attention (Vaswani et al., 2017) is the key to fusing the dual-flow network features. Attention completes the learning of different feature weights, which simply means that higher weights are given to the important features. Suppose we have the two features *F*1 ∈ *R*^*n*×*d*^ and *F*2 ∈ *R*^*m*×*d*^, where **n, m**, and **d** represents a different number of dimensions. *F*1 and *F*2 perform attention such that both vectors have the same dimension **d**. For instance, *Q* is the vector corresponding to *F*1, and *K* and *V* are the vectors corresponding to *F*2. We calculate the attention formula for F1 attention to F2 as follows:


(5) $$\begin{array}{l} Attention\left(Q,K,V\right)=Softmax\left(\frac{QK^{T}}{\sqrt{d_{k}}}\right)V \end{array}$$

where *Q*→*F*1 ∈ *R*^*n*×*d*^, *K, V*→*F*2 ∈ *R*^*m*×*d*^. Finally, the learned attention values are transformed by multilayer perceptron (MLP) and added to F1 to obtain the final feature output:


(6) $$\begin{array}{l} F1^{\prime }=MLP\left(Attention\right)+F1 \end{array}$$

where *F*1′ is the new feature after the attention calculation.

To better learn more features, our model also employs a multi-head attention mechanism. Multi-head attention is expressed as


(7) $$\begin{array}{l} MultiHead\left(Q,K,V\right)=Concat\left(head_{1},...,head_{h}\right)W^{O} \end{array}$$

where


(8) $$\begin{array}{l} head_{i}=Attention\left(QW_{i}^{Q},KW_{i}^{K},VW_{i}^{V}\right). \end{array}$$

We perform feature fusion in stages 3–5 using the multi-head mechanism, where *Layer* = 4, *H* = 4, and *D*_*token*_ = 512 correspond to the number of attention layers, the number of attention heads, and the dimensionality of features, respectively. Note that the outputs of the perception network and motion network have different feature dimensions. To unify the feature dimensions, we adjust the features of the motion network by 1 × 1 convolution after doing the attention calculation, and then we adjust the original number of channels. Let us take stage 3 of feature fusion as an example. The progress is expressed as the formula *MF*′ = *Conv*(*Attention*(*Conv*(*MF*, 512), *PF*), 16), where *PF, MF, andMF*′ represent the feature of the perception network, the feature of the motion network, and the new feature after doing attention calculation, respectively. The attention calculation of the motion network also adds the position of coding information, which is crucial for motion detection because different sequences of pictures represent different movements.

Finally, the features *M*_*o*_ of the motion network perform the deconvolution operation $M_{o}^{{}^{\prime }}=DeConv\left(M_{o}\right)$, which is then summed with the output *P*_*o*_ of the perception network, outputting a 512-dimensional vector of the dual-flow network $Net_{o}=Flatten\left(AvgPool\left(M_{o}^{{}^{\prime }}\right)+AvgPool\left(P_{o}\right)\right)$. Also referring to Codevilla et al. (2019), we map the velocity of the vehicle by the MLP into a 512-dimensional vector and add it to the output *Net*_*o*_ of the network. Then, the output vector dimension remains the same, at 512 dimensions.

Waypoint regression uses the feature vector *Net*_*o*_ of the dual-flow network as input to perform the prediction of waypoints and velocities by an MLP containing two hidden cells < 512, 64>, labeled as *Head*. As the navigation command *C* forms three mutually exclusive values, it also forms three mutually exclusive branches *Head* ∈ {*Left, Right, Straight*}.

#### 3.2.3. Loss function

The model uses the *L*1 loss function MAE to regress speed and waypoints. *W*_*i*_ and $W_{i}^{*}$ denote the ground-truth waypoint and the predicted waypoint, respectively. *V*_*i*_ and $V_{i}^{*}$ denote the ground-truth speed and the predicted speed, respectively. The loss function is denoted as


(9) $$\begin{array}{l} L=\underset{i=1}{\sum }\text{λ}|W_{i}^{*}-W_{i}|+\left(1-\lambda \right)|V_{i}^{*}-V_{i}| \end{array}$$

where λ denotes the balance coefficient of velocity and waypoints, and we choose λ = 0.8, which increases the focus on forecasting waypoints.

### 3.3. PID controller

The proportional-integral-derivative controller fulfills the mapping of waypoints to the low-level controller of the vehicle. We use a longitudinal controller and a lateral controller, where the longitudinal controller outputs the throttle command, and the lateral controller completes the steering command.

The longitudinal controller fits the gap between the average speed of the waypoint and the current speed of the vehicle. The waypoints predicted by the model are denoted as $W^{*}=\left\{W_{1}^{*},W_{2}^{*},\ldots ,W_{k}^{*}\right\}$, and the average speed of the waypoint is


(10) $$\begin{array}{l} V_{t}^{*}=\frac{1}{K}\overset{K}{\underset{k=1}{\sum }}\frac{∥W_{i}^{*}-W_{i-1}^{*}∥}{\delta t} \end{array}$$

where δ*t* represents the time interval between waypoints, and *W*_0_ = 0. The goal of the controller is to minimize the difference between the vehicle's current speed with $V_{t}^{*}$. When the value of the subtraction of the two is negative or the speed is less than the threshold value ϵ, it means braking. In our model, *K* = 4 and ϵ = 20*km*/*h*.

**Figure 4:**
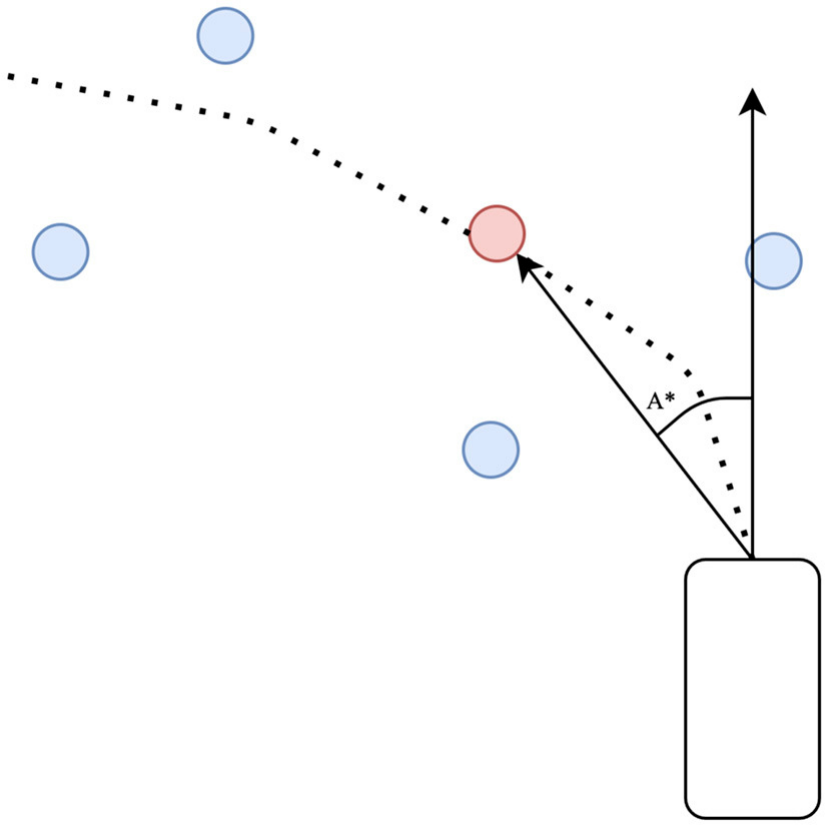
Lateral PID controller. The red waypoint is obtained by fitting a curve with four blue predicted waypoints. A^*^ is the target steering angle.

The lateral controller fits the angle of the waypoint to the angle of the vehicle, as shown in Figure 4. We first fit an arc to four waypoints and steer toward a red point P^*^ on the arc. The goal of the lateral controller is to minimize the angle of the head of the vehicle to P^*^. The final result is


(11) $$\begin{array}{l} A^{*}=tan_{-1}\frac{P_{y}}{P_{x}} \end{array}$$

where A^*^ is the target steering angle.

## 4. Experiments

This chapter details the establishment of the experiments, the comparison of the results, and the related ablation experiments.

### 4.1. Experiment settings

#### 4.1.1. Data collection

Data collection was done in eight cities through the CARLA simulator, version 0.9.12, *via* autopilot mode. City [1 − 4, 6 − 8] was used for training, and city 5 was used for testing. For each city, 10 h of data collection were obtained, with 100 random pedestrians and 70 vehicles. Each episode consisted of start and end coordinates (GPS), and a vehicle at the destination of the limited time without collision means tasks success; otherwise, it means failure. However, if the vehicle infringes on the traffic light rules and does not cause a collision, it also means success. During the data collection, we also added steering noise to enhance the generalization of the data. The collected data contained the RGB video data of 20 HZ from the camera on the front of the vehicle and sensor data including speed, high-level navigation commands, waypoints, vehicle position, head angle, throttle, brake, and acceleration. We removed the effect of weather conditions and used only clear noon weather, considering that the goal of the experiment was to test the performance of the vehicle in complex environments and dynamic objects. The evaluation of the model was performed in city 5 with a set of 10 sub-tasks. Each task contained a starting point, a destination point, high-level navigation commands for the task, a travel length of 1,000–2,000 m, and the yielding of 100 pedestrians and 70 vehicles at predefined locations. The evaluation metrics included route completion (RC), count of collisions (Collision), and time-outs.

#### 4.1.2. Data augmentation

Previous studies Laskey et al. (2017) and Codevilla et al. (2019) have shown that data augmentation is crucial for IL. Therefore, data augmentation was applied to our model at the time of data collection and model training, respectively. During data collection, we injected noise (steer, throttle, and brake) and then returned to normal through the vehicle *via* autopilot. For model training, we cropped the input images with the vehicle as the bottom center, then performed a uniform rotation angle of [−10, 10], and finally performed a random uniform pixel shift [−5, 5] pixels. We also used conventional image enhancement techniques, including Gaussian blur, Gaussian noise, salt-and-pepper noise, and region dropout.

#### 4.1.3. Model comparison

Only models that use images as input were selected for comparison. The CILRS (Codevilla et al., 2019) model uses images from the face-on camera as input, and the ResNet34 backbone network is used for feature extraction, which predicts the vehicle's low-level commands. For comparison with our model, the prediction head of the CILRS model was replaced, labeled as CILRS-W, which can also be seen as using only our perception network for prediction. LBC (Chen et al., 2020) takes images as input and uses a knowledge distillation approach to optimize the “student” network from the “teacher” network. In the latest version, an image heat map is applied to improve its performance. GRIAD (Chekroun et al., 2021) is a video-based autonomous driving algorithm that uses deep reinforcement learning. TCP (Wu et al., 2022) explores the combination of trajectory planning and direct control and ranks second in the CARLA Autonomous Driving Leaderboard. We reproduced it according to the latest code.

#### 4.1.4. Training details

The model uses four multi-head attention and four attention heads for each feature layer, and the input of each attention is the same as that of the perception network feature dimension. Note that the initial value of the input channel of the motion network is equal to the average value of the AlexNet channels. All network models use image enhancement techniques. The ResNet networks were all initialized using AlexNet (Krizhevsky et al., 2012) weights, with an initial learning rate of 0.0001 using the Adam optimizer, and we divided the learning rate by 10 when we found that the network error was not decreasing. Finally, we trained the models with 4 RTX 3090 GPUs for 10 h and a batch size of 12 because of the high resolution of the image of 400 × 176 pixels.

### 4.2. Comparison with model results and analysis

The training and testing results of the model are shown in Table 2. First, our model has the highest success rate. The test task contains numerous pedestrians and vehicles, so LBC cannot detect dynamic objects effectively with the lowest success rate. CILRS-W uses the prediction of waypoints and speed, which improves the model's performance, but it performs poorly in long-distance tasks and when facing the sudden intrusion of dense pedestrian and vehicle traffic. Our model can be seen as integrating the advantages of both the CILRS-W model and the motion prediction network. In the test, the success rate improved by 5%, and the collision rate decreased by 5% compared to CILRS-W. GRID achieves a success rate comparable to that of our model, which uses a deep reinforcement learning approach, while our model uses a supervised learning approach, and the two types of approaches are not directly comparable. TCP is a powerful model that works best by combining the use of trajectory planning and direct control. However, our model explores the extraction of space–time features, and the focus of our study is different from that of the TCP model. Second, the model of migration is better. Compared with CILRS-W, the transfer of the success rate is improved by 2%, and the collision rate is reduced by 3% (19 and 17% for CILRS-W and 17 and 14% for our model). Finally, our model is relatively cautious compared to CILRS-W. The LBC network does not predict vehicle speed, so the time-out is high relative to the task, and the model has a greater tendency to predict speed to 0. CILRS-W uses speed prediction as an aid to reduce time-out cases. However, our model can be seen as CILRS-W overlapping with the motion network branch so that the model is relatively cautious, and the time-out situation is 1% higher compared to CILRS-W.

**Table 2:** Model performance comparisons.

**Method**	**Train**			**Test**		
**-**	**RC**↑	**Col** ↓	**Time-out**↓	**RC**↑	**Col** ↓	**Time-out**↓
LBC	56	36	8	37	63	16
CILRS-W	88	9	3	69	26	5
Ours(w/o A)	90	7	3	71	25	4
Ours	91	7	2	74	21	5
GRIAD	-	-	-	75	20	5
TCP	95	3	2	78	18	4

The table reports the percentage of each metric, and the arrow indicates the direction of the metric better. RC denotes the success rate, and IC is the number of collisions. Our model is safer and has a higher completion rate, although the time-out metric is slightly higher.

**Figure 5:**
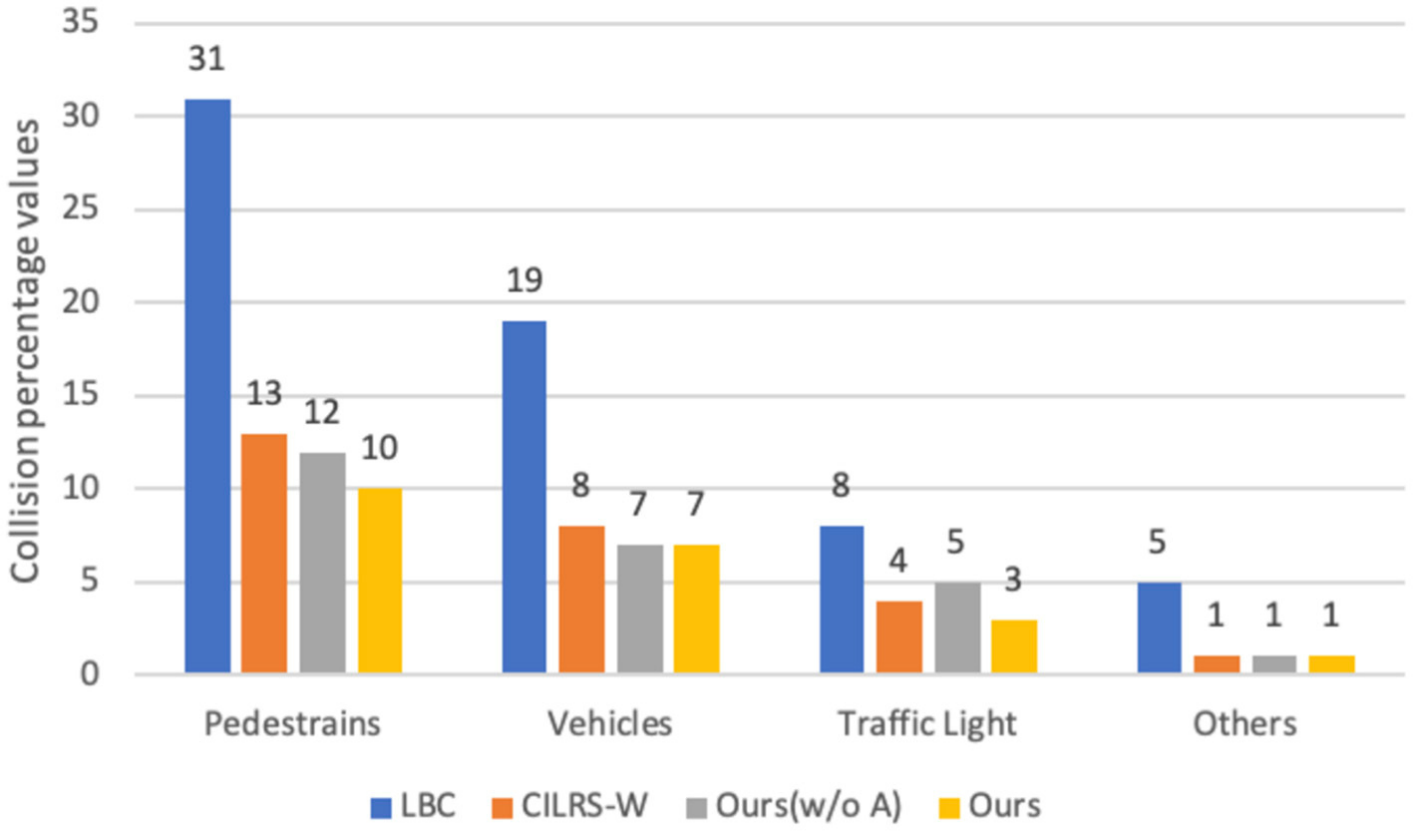
Collision chart. The graph shows the percentage of collisions of each model with pedestrians, vehicles, traffic lights, and others. Our models as shown in yellow have the lowest collision rate.

We further analyzed the collisions in the test, as shown in Figure 5. The LBC model is the least effective. Compared to the CILRS-W model, our model decreases the pedestrian collision rate, the vehicle collision rate, and the traffic light collision by 3, 1, and 1%, respectively. This is because the perception network maintains high-resolution features that help in the recognition of pedestrians, vehicles, and traffic lights; moreover, the motion network estimates the motions of pedestrians and vehicles, which complement each other.

### 4.3. Ablation studies

The core designs are mainly for dual-flow networks and features fusion using an attention mechanism. The driving performance of the dual-flow network is demonstrated in Table 2. To further verify the validity of the model components, we conducted a series of ablation experiments.

#### 4.3.1. Is the attention mechanism valid?

The attention mechanism of the network was removed, and the features of the two networks were summed backward and fused, labeled as ours (w/o A). The results are shown in Table 2. The (w/o A) model decreased the success rate by 3% and increased the collision rate by 4% compared to our model, proving the effectiveness of the attention mechanism. However, too high a level of feature fusion is not beneficial to the alignment of network features.

#### 4.3.2. Are multiple attention layers needed?

The model used the same number of attention layers for each layer of features, initialized each time using Alex weights, trained for 24 h on the training data set, and then validated on the test set. The results are shown in Table 3. It can be seen that the training error of the model tends to decrease as the number of layers increases, and the reason for this trend is related to the increase in the parameters of the model, resulting in a better fit to the training dataset. In the test dataset, the performance of the other models is comparable, except for the first model, whose performance is considered poor. However, the poor migration effect of the model using eight attention layers may be related to the overfitting of the data. In terms of the overall consideration, using four attention layers is the best choice. Note that using a different number of layers for each layer produces better results.

**Table 3:** Attention layer test.

**Attention layer**	**Train RC**	**Test RC**
1	87	72
4^*^	90	75
6	91	75
8	94	73

Train RC and test RC denote the percentage of completed routes on the training and test sets, respectively. Considering the real-time and completion rate, we used four attention layers, marked by ^*^.

#### 4.3.3. Is the ratio of the image sampling rate, marked as α of the dual-flow network, reasonable?

For the purpose of verifying the effect of α (in Equation 4), we used the model with four attention layers for scratch training, and the results are shown in Table 4. α takes too small a value, the motion network cannot learn the motion features, and the whole model degenerates into the CILR-W model. Moreover, α takes too large a value, resulting in too long a period, which is not conducive to the alignment of dual-flow network features and reduces the performance of the model.

**Table 4:** Impact of sampling rate on the dual-flow network.

**α**	**Train RC**	**Test RC**
2	86	70
8^*^	90	73
16	83	69

α refers to the ratio of the sampling rates of perception network and motion network inputs. Train RC and Test RC denote the percentage of completed routes on the training and test sets, respectively. α marked by * is equal to 8, which is the best result and the final choice for our model.

#### 4.3.4. Attention visualizations

**Figure 6:**
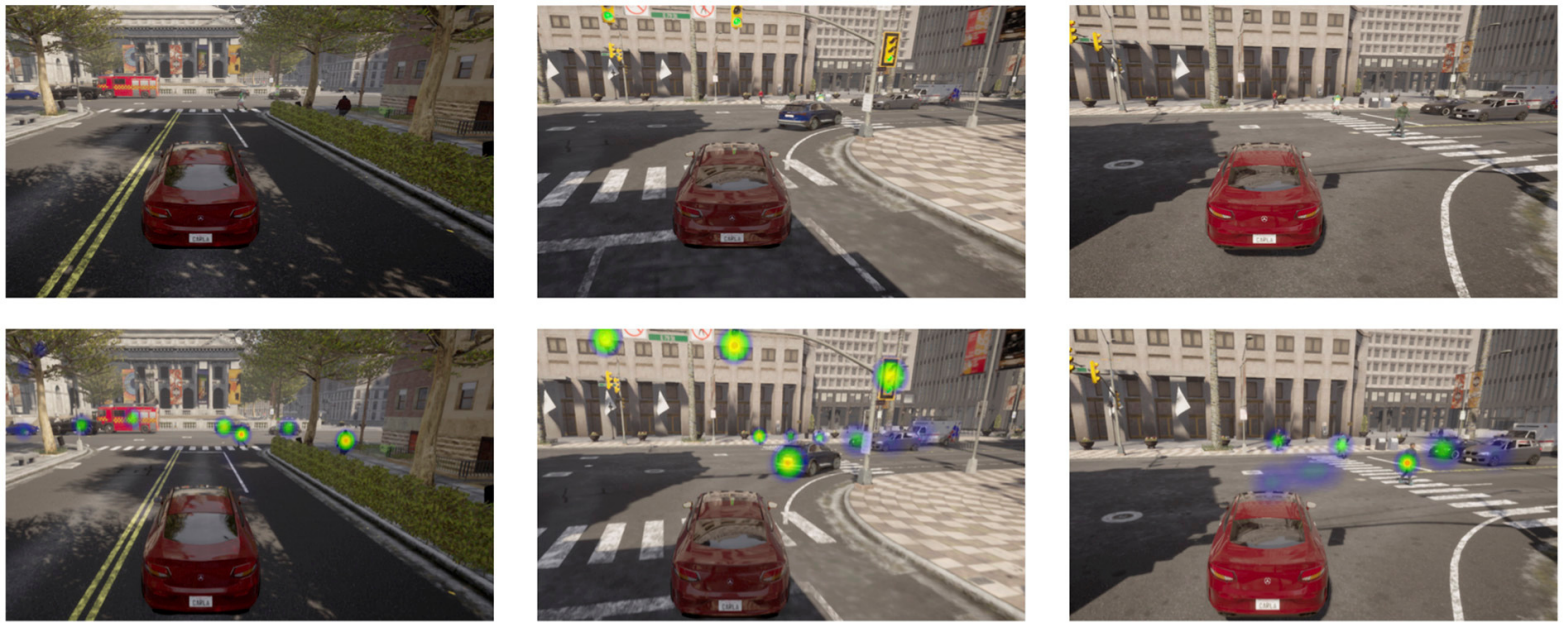
Visual attention. The first row is the original image. The second row is the attention image. The dual-flow network focuses on areas near vehicles, pedestrians, and traffic lights.

The attention mechanism of the network was visualized, as shown in Figure 6. The network has a high weight for moving pedestrians, cars, and signals, which provides help for dynamic object detection in complex environments.

### 4.4. Run time

We measured the running time of our model on a single RTX 3090 GPU by averaging over all time steps of the evaluation route, as shown in Table 5. Some of data in Table 5 are quoted from work (Chitta et al., 2022). Our model takes 35.3 ms per frame, an increase of 11.8 ms and 7.7 ms compared to LF(23.5 ms) and TF(27.6 ms), respectively. Mining the temporal features of the video naturally consumes more time, following our estimation.

**Table 5:** Run time.

**Method**	**Late fusion (LF)**	**Geometric fusion (GF)**	**TransFuser (TF)**	**Dual-flow (Ours)**
MS/Frame	23.5	43.5	27.6	35.3

We obtained the time taken by each model to process a single frame using the total time taken by the model to finish a route divided by the total number of frames. Our model takes 35.3 ms per frame and is relatively time-consuming.

### 4.5. Limitations

First, our best model can learn by imitating the driving habits of an expert at the same level as the expert, which is the ceiling of the model. The expert data bring bias in the data distribution, which is a drawback of our model. Second, the real-time performance of our model needs to be further optimized, as analyzed in other work Section 4.4.

## 5. Conclusion

Motivated by the asymmetry of spatial and temporal sensitivity in the studies of the retinal cell, we proposed a dual-flow network to learn the space–time features of videos for autonomous driving. In the model, the perception network uses a modified ResNet34 backbone to maintain high image resolution and achieve a refined understanding of the environment, while the motion network uses a reduced channel ResNet34 backbone (the channels are reduced to $\frac{1}{8}$), which improves the computational speed and completes the learning of motion information. Finally, the feature layers of two networks are fused using an attention mechanism. Through experiments, we demonstrated that the network structure we designed aided in the detection of dynamic objects in complex environments, achieving a completion rate of 74%.

In the future, we will try to use deep reinforcement learning to relieve the distribution mismatch caused by IL and to increase the adaptability to unknown scenarios. Also, the real-time performance of our model needs to be further optimized. Inspired by recent research (Shang et al., 2022; Yuan et al., 2022a,b), autonomous driving perception algorithms can also be used to extract low-dimensional information relevant to decision-making from high-dimensional information. The application of multi-sensor data, especially video data (high latitude data) and LiDAR point cloud data (sparse data), is a new direction worthy of research. Hopefully, our research will promote the use of space–time features in autonomous driving.

## Data availability statement

The raw data supporting the conclusions of this article will be made available by the authors, without undue reservation.

## Author contributions

LY, WL, WZ, and TY contributed to conception and design of the study. TY organized the database. LY, WL, and WZ performed the statistical analysis. LY wrote the manuscript. All authors contributed to manuscript revision, read, and approved the submitted version.
